# Collectanea Medica, Consisting of Anecdotes, Facts, Extracts, Illustrations, Queries, Suggestions, &c.

**Published:** 1819-04

**Authors:** 


### COLLECTANEA MEDICA,

CONSISTING OF

### ANECDOTES, FACTS, EXTRACTS, ILLUSTRATIONS, QUERIES, SUGGESTIONS, &c.

RELATING TO THE

***History or the Art of Medicine, and the Auxiliary Sciences.***

Quicquid agunt medici, nostri farrago libelli.

### *Medical Knowledge of the People of the Tonga Islands.*

*(Continued from page 214.)*

THE most common surgical operation among the Tongese is what they call *tafa*, which is topical blood-letting, and is performed by making, with a shell, incisions in the skin to the extent of about half an inch in various parts of the body, particularly in the lumbar region and extremities, for the relief of pain, lassitude, &c.; also for inflamed tumors, they never fail to promote a flow of blood from the part. By the same means they open abscesses, and press out the purulent matter. In cases of hard indolent tumors, they either apply ignited tapa or hot bread-fruit repeatedly, so as to blister the part, and ultimately to produce a purulent surface. Ill-conditioned ulcers, particularly in those persons whose constitution disposes to such things, are scarified by shells; those that seem disposed to heal are allowed to take their course without any application.

In cases of sprains, the affected part is rubbed with a mixture of oil and water, the friction being always continued in one direction,—that is to say, from the smaller towards the larger branches of the vessels. Friction, with the dry hand, is also often used in similar and other cases, for the purpose of relieving pain.

In respect to inflammations of the eyes, which sometimes rise to a very great height, attended frequently with a considerable purulent discharge, they frequently have recourse to scarification by the application of a particular kind of grass,—the minute spicula with which it is replete dividing the inflamed vessels, as it is moved upon the tunica adnata. To assist in reducing ophthalmic inflammations, they also drop into the eye an acid vegetable juice, and sometimes another of a bitter quality; the first is called *vi*, the latter *bawlo*. The species of ophthalmia to which they are subject, though sometimes lingering, is stated scarcely ever to have produced serious consequences, and is not considered contagious. Mr. Mariner neither saw nor heard but of one man who had lost his sight by disease.

In cases of gun-shot wounds, their main object is to lay the wound open, if it can be done with safety in respect to the larger blood-vessels and tendons; not only for the extraction of the ball, if it should still remain, but for the purpose of converting a fistulous into an open wound, that it may thereby heal sooner and better. If they have to cut down near larger vessels, they use bamboo in preference to the shell; the same near tendons, that there may be less chance of injuring them. They always make incisions nearly in the course of the muscles, or at least parallel with the limb.

The amputation of a limb is an operation very seldom performed; nevertheless, it has been done in at least twelve individuals. Mr. Mariner seeing one day a man without an arm, curiosity led him to enquire how it happened, and found that he had been one of the twelve principal cooks of Toogoo Ahoo, the tyrant of Tonga, and had submitted to the amputation of his left arm, under the circumstances related in vol. i. p. 76. The mode in which this operation was performed was similar to that of *tootoonima*, described vol. ii. p. 222; only that a large heavy axe was used for the purpose. The bleeding was not so profuse as might be imagined; owing, no doubt, to the bluntness of the instrument and violence of the blow. This stump appeared to Mr. Mariner to be a very good one; the arm was taken off about two inches above the elbow. Ten were stated to have done very well; of the remaining two, one died of excessive hæmorrhage, and the other of mortification. There was also a man, living at the island of Vavaoo, who had lost a leg in consequence of the bite of a shark, which is not a very uncommon accident; but there was something unusual in this man's particular case: his leg was not bitten off, but the flesh was almost completely torn away from about five inches below the knee down to the foot, leaving the tibia and fibula greatly exposed, and the foot much mangled. He was one of those who chose to perform his own operations; with persevering industry, therefore, he sawed nearly through the two bones with a shell, renewing his tedious and painful task every day till he had nearly accomplished it, and then completed the separation by a sudden blow with a stone! The stump never healed. Mr. Mariner had this account from the man himself and many others.

*Téfe*, or the operation of circumcision, is thus performed:—a narrow slip of wood, of a convenient size, being wrapped round with gnatoo, is introduced under the præputium, along the back of which a longitudinal incision is then made to the extent of about half an inch, either with bamboo or shell (the latter is preferred): this incision is carried through the outer fold, and the beginning of the inner fold, the remainder of the latter being afterwards torn open with the fingers; the end of the penis is then wrapped up in the leaf of a tree called gnatái, and is secured with a bandage: the boy is not allowed to bathe for three days. The leaf is renewed once or twice a day. At the Fiji islands, this operation is performed by amputating a portion of the præputium, according to the Jewish rite.

They are very subject to scrofulous indurations, glandular enlargements, and ulcers: they call the disease *cahi:* the parts affected are the groins, axillæ, and neck; though many other parts of the body are also liable to ulcers, which they call *palla.* These diseases sometimes run on to such an extent, and assume such appearances, that we believe some travellers have mistaken them for lues venerea. It is certain that some individuals affected with palla have been obliged to submit to the loss of a nose, the cartilagenous and softer parts of that organ becoming completely destroyed: it must be also mentioned, at the same time, that the natives are subject to gonorrhœal discharges, attended with ardor urinæ. All these circumstances appear very equivocal: but Mr. Mariner has every reason to believe that the venereal disease did not exist under any form, either at the Hapai islands or Vavaoo, during the time that he was there; although, to his certain knowledge, three of the survivors of the Port-au-Prince's crew had gonorrhœas at the time the ship was taken; one of whom had brought it from England, and the other two had contracted it at the Sandwich islands. Several others of the ship's company had also venereal affections; but they fell in the general massacre on-board. In the first place we must observe, in respect to those labouring under the diseases called cahi and palla, that the complaints are either not venereal, or that the venereal disease subsides in them, and the constitution cures itself spontaneously. 2dly, That the organs of generation are never affected previously to the more general disease coming on. 3dly, That these diseases are not known to be, or believed to be, contracted by sexual intercourse. 4thly, That though these diseases, in some constitutions, produce fatal consequences, yet very frequently the appetite and strength, and fullness of flesh, remain much the same as if no disease existed; though this happens in palla more than in cahi. In respect to the gonorrhœas to which they are subject, they are for the most part very mild in their symptoms, and get well in a few days; besides which, they are not capable of being communicated between the sexes,—or, at least, this is not known or believed to be the case. In regard to the three men of the Port-au-Prince's crew, they got well without exactly knowing when or how; for the consternation occasioned by the capture of the ship and the destruction of their countrymen, and the alarm and state of anxiety in which they were for at least two or three days, had produced such a change in the constitution, or at least in the disease, that it had actually got well before they were aware of it. Mr. Mariner enquired among some of the older men, if they had ever seen or heard of such a disease as syphilis or venereal gonorrhœa, (describing the general character of it, and how it was communicated;) and learnt that a woman, a native of one of the Hapai islands, having had connexion with one of the men belonging to a French ship, became on fire (as they expressed it), and died afterwards in a very bad state: and this was all that he learned respecting what might reasonably be supposed to be true syphilis. Palla frequently gets well spontaneously; but the remedies commonly used are scarification of the ulcered surface, powder of turmeric sprinkled over it, and sometimes a bitter vegetable juice dropped on it.

They have among them another kind of ulcerous disease, which they call *tona*, very distinct from the two last described, children being for the most part subject to it; and it is one of those diseases which only occur once during a person's life. The patient is first seized with general languor and debility, attended with loss of appetite: in a few days an eruption appears in different parts of the body, but particularly in the corners of the mouth, axillæ, groins, parts of generation, and anus; the pustules at first are exceedingly small, but at length increase to about half an inch in diameter; fungous excrescences grow out of them, exhibiting a granulated surface, and discharging a viscous fluid, which concretes round the edges. These pustules come also upon the soles of the feet, and increase to a considerable size, giving very great pain. Mr. Mariner is not acquainted with the state of the pulse, &c. The disease generally lasts several months, and sometimes a couple of years. From the symptoms thus far described, there is not much doubt about its resemblance to what is called the *yaws:* the remedies they use for it are a certain bitter juice dropped into the ulcers, and rubbing off the fungous excrescences with cocoa-nut husk dipped in sea-water. They are subject also to a pustulous eruption, chiefly confined to the feet, but which sometimes affects the hands. It usually appears between the toes, and has in its external character a strong resemblance to psora, and itches very much: it appears in the form of small pustules with whitish heads, which, when rubbed off, generally discharge a watery fluid. It is supposed to arise from walking in clayey places without the opportunity of washing the feet afterwards: it is not thought to be contagious; it usually lasts about four or five days. The name they give it is *gnöwooa*. They use no remedy.

They are also subject to a disease called *fooa:* but, if we describe the symptoms of elephantiasis, we shall have related with tolerable accuracy the history of this disorder. Labillardiere notices the disease, and calls it elephantiasis. They use no remedy for it.

The disease called *momoco* usually lasts from four to seven months: in the latter stages it somewhat resembles phthisis. It comes on with occasional chilliness, loss of appetite, lowness of spirits, wasting of the flesh: shortly succeed swellings in the groin and axillæ, general debility, paleness of the lips. As the disease advances, the patient stoops very much, experiences pains in the chest, and across the shoulders; sometimes, but not often, a cough, and expectoration now supervene, the debility and emaciation become extreme, and death relieves the patient from his sufferings. These are all the symptoms which Mr. Mariner can speak of with certainty. They use no physicial remedies.

*Féke-féke* appears to be a sort of mild irregular intermittent: the paroxysm usually lasts from two to eight hours, and consists of a cold and a hot stage; but is seldom succeeded by perspiration. The returns of the paroxysm are very uncertain: sometimes two, at other times three, four, five, or more days intervene. The patient is sometimes perfectly well for a month, and then his disorder returns.

In regard to diseases properly belonging to females, Mr. Mariner has very little to communicate. The women are in general tolerably healthy. During the catamenia, they anoint themselves all over with a mixture of oil and turmeric, to avoid catching cold; and they do the same after lying-in; on which occasions women always assist, to the perfect exclusion of the other sex. Respecting the circumstances of parturition, and the separation of the child, these things are kept a profound secret from the men. The men also occasionally use this mixture of turmeric and oil in time of war, when the weather is wet, to prevent them from feeling chilly, for at that time they have scarcely any dress. Mr. Mariner on similar occasions has anointed himself all over with it, and found it to have the desired effect.

---

On looking over the *Dictionnaire des Sciences Medicales*, for the purpose of selecting from it such brief disquisitions as are appropriate for our *Collectanea*, our attention was arrested by an article entitled *Cas Rares*, written by M. Fournier. Rare and curious cases, which have not furnished matter for any immediate induction of utility to physiology or the practice of medicine, have generally been neglected in the records of this science, or only noticed by some more inquisitive writers, who have collected them together in compilations that have themselves usually been buried in the libraries of the curious. The *Ephem. Erudit.*—the *Ephem. Nat. Curios.*—the Swedish *Amœnitates Academicæ*,—the *Philosophical Transactions* of London,—the *Mem. de l'Acad. des Sciences* of Paris,—the *Nov. Act. Nat. Curiosorum*,—the Hierne *Collect. Academicæ*,—the *Commentaries* of Van Swieten,—the *Erst Grunde eines Physiologie der eigentlichen thierischen Naturthierischer Koerper*, of Unzer,—and the *Magazine zum Erfahrungfseelenkunde*,—contain many cases of this kind that merit the attentive consideration of physiologists, now that the modern discoveries in that branch of science will render intelligible many phenomena that presented a mere chaos to the minds of those who recorded them. We must not omit to mention the *Physiological Nosology* of Mr. Good amongst the sources whence much curious and valuable matter of this kind may be derived: that work, not considering its chief object, is highly valuable from the numerous collection of interesting cases related in it or referred to, and for this reason alone it merits a place in the library of every liberal student of medicine. We consider that the thanks of the profession are due to M. Fournier, for having undertaken to write the history of rare and curious cases: it was an arduous task, and not likely to meet with the reward due to the labour required to effect it. The present article occupies the space of a hundred pages, and is said by the author to be "rather a rapid sketch than a complete history. If, however, the patronage of some part of the profession is not refused to this labour, I shall pursue it, and shall profit by the materials that I have collected to compose a more complete essay on rare cases." We shall select from this work the principal part of such facts as coincide with the views we have disclosed, and shall commence with the subject of *Uterine Conception.*

"The annals of the history of man are filled with extraordinary facts of the aberrations of nature from the ordinary state in the phenomena of conception; I shall confine myself to the recital of a few of the most interesting and remarkable.

Amédea Bissieu complained, from his infancy, of a pain in the left side: this side was larger than the opposite one, and presented a tumor which gradually increased in size. The physical and moral faculties of the child nevertheless became well developed. When about thirteen years of age he was suddenly attacked with fever, and the tumor became voluminous and more painful. After the lapse of some days the patient voided by stool a quantity of fetid, puriform, matter. Three years after this attack, he became affected with pulmonary phthisis; and at this time he voided by stool a ball of hair. He died about six weeks afterwards, in a state of consumption, at the age of fourteen. Two medical practitioners opened the body: they found in a bag attached to the transverse portion of the colon, and then communicating with the cavity of that intestine, several balls of hair, and an organized mass having many traits of resemblance to a human fœtus: this mass, on careful dissection, discovered traces of some of the organs of sense; a brain, spinal marrow, very voluminous nerves, muscles degenerated into a sort of fibrous matter; a skeleton composed of a vertebral column, with the head, pelvis, and almost the whole of the extremities; an umbilical cord, very short, was inserted into the transverse mesocolon, with an artery and a vein ramified by the opposite extremities in the parts of the fœtus, and on the mesocolon. The organs of digestion, of respiration, and of the urinary section, as well as those of generation, were wanting."

This case, as our readers will perceive, resembles in its principal circumstances that which occurred to the observation of Mr. Highmore, of Sherborne, in the year 1814; an account of which was published by him, and the subject publicly exhibited in London. An analogous instance is also related in the first volume of the Medico-Chirurgical Transactions.

*(To be continued.)*

***Anatomico-Physiological Examination of the Medicinal Leech;*** by Henry Lebrecht Kurzmann, M.D. Physician to his Majesty the King of Prussia, &c. With five Plates.—Berlin, by Stuhr, 1817. 8vo. pp. 107.*

The natural history and anatomical description of this animal has, indeed, been frequently discussed, and has of late amply and judiciously employed the able pen of Dr. Johnson: nevertheless, the following extract from the work of a gentleman, who for a series of years has been engaged in the investigation of the structure and nature of this animal, (which perhaps by no other is surpassed in medical utility,) it is hoped, will not be deemed unacceptable. In the course of his researches, he has succeeded in making some new observations, besides finding many former ones confirmed. Without entering into any farther preliminaries, we shall, therefore, at once proceed to give the substance of the work.

The leech is thickly covered with a gelatinous substance, of a somewhat deeper grey and more aqueous nature than the similar one of snails: this covering penetrates deeply into the skin, and appears to be essentially necessary for the existence of the animal; it soon getting exhausted, and dying on its being repeatedly wiped off. Beneath this covering a thin epidermis is met with, which, bursting in front of the abdomen, is cast off twice or thrice a year, commonly retaining the perfect shape of the body.

Under this epidermis appears a coreaceous coat or cutis, containing those coloured spots that cover the whole leech, and is disposed in rings or annular muscles, that surround it transversely; of which Dr. K. counted ninety-four on the body, and six on the upper lip. The skin of each ring consists of minute, oblong, quadrangular laminæ, of equal size, placed in double rows behind each other, and may be seen particularly plain on the back; their larger edge being situated longitudinally, and the short ones across the body. On the animal's contracting itself, the laminæ of one annular row encounter those of the other of the same ring, though not all in an equal degree, but each single pair, as they are situated in succession, either more or less; giving the skin a tubercular appearance.

The head consists only of an oval lip that projects from the body, the rim of which is bent downwards, and met below by the rim of the body, somewhat bent upwards in the shape of an under-lip. Of these lips the leech makes use, partly for receiving its nutriment, and partly in its advancing motions. On

---

* This abstract of a work on an interesting subject of Natural History was communicated to us by Dr. Von Embden, of Hambourg; and, in justice to the literary acquirements of that intelligent physician, we think it proper to inform our readers that it is given in his own words. It is but rarely that we find our language written with such facility and gracefulness of expression by a foreigner.—Edit.

each side of the first ring of the upper-lip, three small black spots or tubercles make their appearance, elevating themselves in a somewhat globular shape; and two similar ones are seen on the upper rim of the following rings. These spots which, even in the largest leeches and in the clearest light, can be perceived by the naked eye only when the lip is distended lengthways, have erroneously been taken for the eyes of the animal; but our author is of opinion that the leech is entirely destitute of the faculty of sight, and considers these supposed eyes to be mere organs of feeling; which he proves partly by the animal's not showing the least sensibility on its sudden exposure to light, and partly by its motions, as it always bends the lip in the shape of a hoof whenever it is going to quit its place, not previously touching every occurring object with the point of the lip, but rather touching, and in a manner feeling for it, with the sides where the eyes are supposed to be situated.

On the belly, between the 24th and 25th ring, counted from before, a white skinny spot, with a small central cleft, is discovered. This is the orifice of the penis, which, in dead leeches, especially when killed by means of hot water, hangs out in the form of a string, of the thickness of common sewing thread, to the extent of three or four lines.

Farther down, in a straight direction under the said spot, between the 29th and 30th ring, appears a similar spot, which is the opening of the vagina. Besides these two openings, there are, on either side of the belly, on each fifth ring, the apertures for the respiratory organs.

On the back of the leech, in the salone, between the body and the caudal extremity, (or, as our author terms it, the foot,) appears an exceedingly small slender slit, constituting the anus. The foot, or round skinny disk, projecting all round the body like the rim of a plate, consists of a texture of muscular fibres, crossing each other in every direction, and possesses, like the mouth, the capacity of fastening itself by suction to objects with considerable force, and, in conjunction with the mouth, assists the leech, as is well known, in moving forward.

Beneath the cutis is the muscular coat, the muscular fibres of which may be plainly exhibited by fastening a living leech, extended in such a manner as not quite to smooth the rings, to a plate of wax; then killing it quickly by means of boiling water; and, thus prepared, exposing it for some hours to the action of nitric acid, diluted with one-third of water. If now the cutis be carefully dissected and laterally separated from the muscular coat, the latter will be found to consist of a number of fibres, placed one upon another, and running in a straight direction from the oral down to the caudal sucker. They lie thickest on the upper part of the animal, round the œsophagus, where they in some measure appear to take their origin; but appear to grow thinner, and even to lose themselves totally, towards the caudal extremity, particularly at the sides of the animal. Every pair of laminæ in each ring is provided with a separate muscle, which is fastened with its lower end to the subjacent muscle; then running on in a straight line from the forepart backwards, dividing itself at its posterior end into two strings, one of which adheres to the fore-rim of one lamina, and the other to the back of the succeeding one; but, on the rings situated more backwards, it runs on, under twelve, sixteen, or more rings, before it attaches itself to any one. To this disposition it is owing that the leech is capable of making the most different motions, and of assuming the greatest variety of shapes.

This greater number of muscles in the oral region gives the leech its great force of suction, and the smaller number of the same towards the caudal extremity is made up by those of the caudal sucker. In very large leeches, Dr. K. found another layer of muscles just above those described, which disappeared on both sides in the skin of the animal, and ran in a slanting direction across the body; so that each single fibre stretched itself under twenty or more rings. They were exceedingly slender, and firmer than the other muscles; lay not quite so close together; and did not run uninterruptedly all round the body, those on the back being separated from those of the belly.

Below the muscular coat appears another, which Dr. K. calls the villous coat; a fibrous velvet-like substance, of a dark-brown colour, closely covering the whole inner surface of the muscular one, firmly adhering to the same, but no where connected with the inner parts. At the outermost rim of the upper and under lip, a thin white single coat takes its beginning, and is to be considered as a continuation of the epidermis, lines the inner surface of the lips. The two folds situated behind the upper-lip, then turning over the three rows of teeth, lines the inner surface of the œsophagus, and proceeds to form the alimentary canal. The two folds just mentioned are of a semilunar shape, and of a solid, though less firm consistence than the external coat. The foremost one is commonly larger than the hindermost. They seem to be peculiarly useful to the leech in its advancing motions by means of the mouth, then covering the teeth, which otherwise would prove a hindrance in these exertions.

Behind these semilunar folds the œsophagus takes its commencement, which, during the extension of the leech, is about four lines long and two broad, consisting of thick pretty firm muscular fibres, that appear to run in layers, nearly in a slanting direction, from the top to the bottom, and partly from the belly to the back in a straight line, joining each other at their hinder end. Some fibres do, however, also stretch themselves more than an inch over the alimentary canal, without joining the same, losing themselves in the general muscular coat. By means of this double layer of muscular fibres, the œsophagus is rendered capable of contracting itself both longitudinally and latitudinally.

*(To be continued.)*

